# Rapid Accumulation of Mutations in Growing Mycelia of a Hypervariable Fungus *Schizophyllum commune*

**DOI:** 10.1093/molbev/msaa083

**Published:** 2020-04-06

**Authors:** Aleksandra V Bezmenova, Elena A Zvyagina, Anna V Fedotova, Artem S Kasianov, Tatiana V Neretina, Aleksey A Penin, Georgii A Bazykin, Alexey S Kondrashov

**Affiliations:** m1 Center of Life Sciences, Skoltech, Moscow, Russia; m2 Surgut State University, Surgut, Russia; m3 Belozersky Institute of Physico-Chemical Biology, Lomonosov Moscow State University, Moscow, Russia; m4 Kharkevich Institute for Information Transmission Problems, Moscow, Russia; m5 Vavilov Institute of General Genetics, Moscow, Russia; m6 N. A. Pertsov White Sea Biological Station, Lomonosov Moscow State University, Primorskiy, Russia; m7 Department of Ecology and Evolutionary Biology, University of Michigan, Ann Arbor, MI

**Keywords:** mutation rate, somatic mutation rate, *Schizophyllum commune*

## Abstract

The basidiomycete *Schizophyllum commune* has the highest level of genetic polymorphism known among living organisms. In a previous study, it was also found to have a moderately high per-generation mutation rate of 2×10^−8^, likely contributing to its high polymorphism. However, this rate has been measured only in an experiment on Petri dishes, and it is unclear how it translates to natural populations. Here, we used an experimental design that measures the rate of accumulation of de novo mutations in a linearly growing mycelium. We show that *S. commune* accumulates mutations at a rate of 1.24×10^−7^ substitutions per nucleotide per meter of growth, or ∼2.04×10^−11^ per nucleotide per cell division. In contrast to what has been observed in a number of species with extensive vegetative growth, this rate does not decline in the course of propagation of a mycelium. As a result, even a moderate per-cell-division mutation rate in *S. commune* can translate into a very high per-generation mutation rate when the number of cell divisions between consecutive meiosis is large.

## Introduction

Mutation rate is a key parameter of evolution. Fortunately, the development of next-generation sequencing technologies made it possible to measure mutation rates directly, by comparing genotypes of parents and offspring, and data on species from a wide variety of taxa are accumulating rapidly. The majority of nonneutral mutations are deleterious, and it has long been proposed that natural selection usually tries to minimize the mutation rate (Lynch et al. 2016). The per-generation mutation rate in a multicellular organism is a product of the mutation rate per cell division and the number of mitoses between two consecutive meiosis. Thus, the per-generation mutation rate can be modulated by two, not mutually exclusive, mechanisms. The first one is to reduce the per-cell-division mutation rate, and the second one is to reduce the number of mitoses between consecutive meiosis. For brevity, we will refer to them as “fidelity” and “economy” mechanisms, respectively. Both can be implemented in a variety of ways.

The fidelity mechanism can involve reduction of the mutation rate in all cells. However, in this case, the cost of fidelity (Blomberg 1987) is incurred across the board. Thus, in species with a dedicated germline, the per-cell-division mutation rate may be specifically reduced, by as much as an order of magnitude, only in germline cells, as it is the case in mammals (Milholland et al. 2017).

The economy mechanism can also depend on the existence of a dedicated germline, if it is shielded from repetitive divisions during the lifetime of an organism, as it is the case in females, although not in males, of mammals (Kong et al. 2012; Jónsson et al. 2017). However, there are ways to reduce the number of mitoses between consecutive meiosis even in the absence of a germline. First, shoots or hyphae of an organism may possess apical cells that divide only rarely because most of the growth occurs due to intercalary cell divisions (Lanfear et al. 2013; Anderson and Catona 2014). Another potential scenario is an increase of cell size, which reduces the number of cell divisions required for a given amount of linear growth.

Obviously, a reduced per-cell-division mutation rate still leads to a linear accumulation of mutations with the number of mitoses between consecutive meiosis. Similarly, if the economy of cell divisions is achieved by the increased size of cells, mutations still should accumulate linearly with somatic growth of an organism. In contrast, a shielded germline or apical cells are likely to lead to decelerated accumulation of mutations with age or in the course of somatic growth. The number of cell divisions before gametogenesis and, thus, the number of mutations accumulated per-generation was found to be independent of the life span and the extent of vegetative growth in *Arabidopsis thaliana* (Watson et al. 2016), indicating that the economy mechanism is operating, perhaps through intercalary growth.

Mutation rate-reducing mechanisms can be particularly salient in species where individuals can reach huge sizes, such as some plants and fungi. Indeed, in several such species, the number of genetic differences between even remote parts of the same individual is surprisingly low. This is the case for the oak *Quercus robur* (Schmid-Siegert et al. 2017), a giant honey mushroom *Armillaria gallica* (Anderson et al. 2018), and the fairy-ring fungus *Marasmius oreades* (Hiltunen et al. 2019).

Although the mutation rate per-generation can be easily measured by comparing individuals, measuring the mutation rate per cell division is harder. In multicellular organisms, this could be achieved by either direct sequencing of a cell and its offspring, or of two cells separated by a known number of cell divisions. However, single-cell sequencing is still in its infancy, and it is hard to track cell lineages within an individual, which precludes precise estimates of the number of cell divisions separating two locations within the organism.

Mycelial fungi are characterized by linear mycelial growth, possibly simplifying this task. Still, making use of this advantage is difficult. First, the exact linear distance between locations within a mycelium can only be measured in a lab, and many fungi cannot be easily cultivated. Second, it remains unknown how the number of cell divisions scales with the linear distance. Third, fungi often have multinuclear cells, complicating measurements, and interpretation of data.

To better understand the relationship between the somatic accumulation of mutations during growth and their contribution to the per-generation mutation rate, we studied a basidiomycete wood-decay fungus *Schizophyllum commune*. This species is characterized by the highest known within-population nucleotide diversity, which reaches 0.20 (Baranova et al. 2015). The cause of this extraordinary diversity remains unclear. Although the per-generation mutation rate in this species, measured by Baranova et al. (2015), is quite high (2.0×10^−8^ per nucleotide), it is not extreme (Lynch et al. 2016). However, this measurement could possibly underestimate the natural per-generation mutation rate in *S. commune*. Indeed, the measurement of Baranova et al. (2015) was based on cultivating two mycelia on the same Petri dish, which grew, mated, and formed fruit bodies, and a generation involved only ∼10 cm of mycelial growth. In nature, the amount of growth between consecutive meiosis may be much higher, which can result in a much higher per-generation mutation rate. Of course, the extraordinary genetic diversity of *S. commune* can also result from an exceptionally high effective population size.

Here, we present an experimental study of accumulation of mutations in a mycelium of *S. commune* over a long period of linear growth which intends to shed light on the mutation process in this species.

## Results

### Measuring the Mutation Rate in a Linearly Growing Mycelium

The life cycle of *S. commune* includes a mononuclear haploid stage which originates from a single spore (Schmid-Siegert et al. 2017; Hanlon 2018). This stage can be relatively easily cultivated on solid media, where it grows vegetatively without producing fruit bodies. The mycelium of *S. commune* grows linearly and apically in cell-thick hyphae (Gooday 1995); the cell length is known, and comprises ∼100 µm (Essig 1922). Knowing the number of cell divisions between two points of the mycelium, it is easy to estimate the mutation rate per cell division.

We developed an experimental system that allows us to cultivate haploid mycelia of *S. commune* for a long period of time, maintaining strictly vegetative mode of growth and an approximately constant number of growing hyphae. Each culture was started from a single haplospore which gave rise to a haploid mycelium with mononuclear cells (Stankis et al. 1990), and was then cultivated in glass tubes of a fixed diameter on solid medium. We regularly measured growth rates of the mycelia along the tube, and took samples for sequencing. We also sequenced all founding cultures and performed genome assembly for each culture individually. Sequenced samples of derived cultures were then mapped to corresponding assemblies; this was done to achieve good mapping quality, as mapping on an assembly of a different individual is difficult because of the high genetic diversity of *S. commune* (Baranova et al. 2015).

**Fig. 1.**
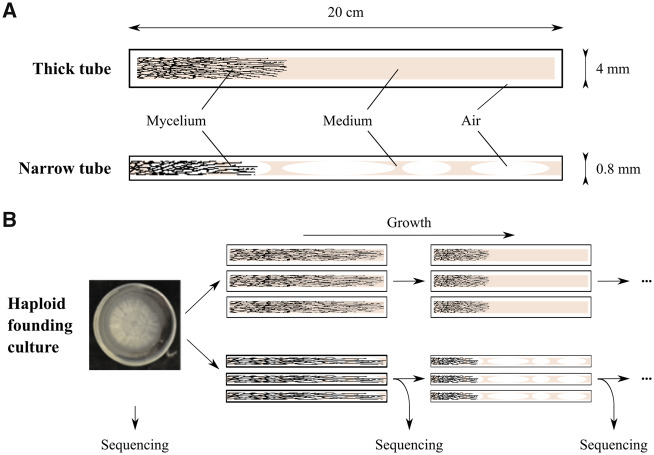
Experimental system. (*A*) Schematic representation of the tubes used in the experiment (not to scale). (*B*) Overall experimental layout.

We used tubes of two different diameters. Narrow tubes had an inner diameter of ∼0.8 mm, with its width partially filled with solid medium. Thick tubes comprised a cylinder of solid medium 4 mm in diameter, placed within a glass tube with a slightly larger inner diameter (fig. 1*A*). The tubes were 15–20 cm long, and cultures were transferred to the next tube as soon as the hyphae reached the end of the tube. In the case of narrow tubes, this procedure by itself did not always yield successful replanting because the number of transferred cells was too small. Therefore, before the transfer to the next tube, cultures were cultivated on Petri dishes for some time to obtain enough material. The overall period of growth on the Petri dish was ∼20 times shorter than that in the tubes, and was not counted toward the overall growth time of the corresponding mycelium. For transfer, we then attempted to sample cells from the same position of the Petri dish where the culture was planted, minimizing the number of mutations accumulated on the Petri dish. At the time of transfer, mycelial samples were also collected for sequencing. The overall experimental layout is shown in figure 1*B*. Measured cell lengths for different cultures and different tube sizes are presented in supplementary table S1, Supplementary Material online; we estimated mean cell length at 163 (95% CI: 154.75–171.25) and 165 µm (95% CI: 157.48–172.52) in narrow and thick tubes, respectively.

### Experimental Lines

We used four founding haploid cultures, each originated from a single haplospore. Three of the cultures (sh01, sh02, sh03; specimen vouchers WS-M203, WS-M222, WS-M276) were obtained from fruit bodies collected in Ann Arbor, MI, and one culture (sh04; specimen voucher WS-M45), from a fruit body collected in Moscow, Russia. Each founding culture was used to start six experimental lines in tubes of two different diameters (three replicates in each), for a total of 24 experimental lines.

**Fig. 2.**
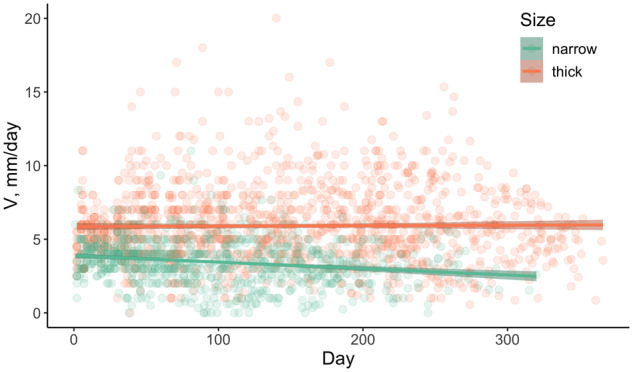
Growth rates in thick and narrow tubes during the experiment. Data for all lines are pooled together. Linear regression for narrow tubes: *R*^2^ = −0.04, *P* value = 3.7×10^−9^. Linear regression for thick tubes: *R*^2^ = 1.2×10^−4^, *P* value = 0.68.

We cultivated them for between 220 and 360 days. The mean growth rate in the thick tubes (5.9 mm/day) was almost twice as high as in the narrow tubes (3.5 mm/day). The cultures have grown up to 96 cm in narrow tubes, with the mean 78 cm (corresponding to ∼4,800 cell divisions), and up to 247 cm in thick tubes, with the mean 198 cm (∼12,000 cell divisions). The growth rate remained constant in the thick tubes, whereas in the narrow tubes, it decreased slightly but significantly (fig. 2).

### Accumulation of De Novo Mutations

**Fig. 3.**
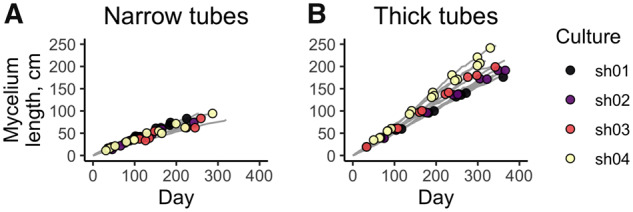
Growth of the mycelia during the experiment in narrow (*A*) and thick (*B*) tubes. Sequenced points are marked with circles.

We obtained and sequenced a total of 112 samples of growing mycelium. Each of the 24 lineages was successively sampled from four to seven times (fig. 3). Each sample was sequenced with the average coverage 135×, and a total of 300 de novo mutations was detected (supplementary table S2, Supplementary Material online); the mutational spectrum is shown on supplementary figure S1, Supplementary Material online. Among these mutations, 63 were coding, including 45 nonsynonymous mutations, 2 nonsense mutations, 3 frameshifts, and 1 stopgain insertion. Most of these mutations were fixed in the mycelium, that is, have been present in all or nearly all reads in all subsequent time points; still, a number of mutations have reached high frequencies but were later lost, and some mutations have never reached high frequencies (table 1). In each line, the vast majority of mutations that were observed at the last time point (72–100%) were fixed. We observed no parallel mutations between different founding cultures. Among coding mutations, the overall d*N*/d*S* ratio was somewhat lower in thick tubes (0.7) than in narrow tubes (1.0), although the difference was not significant.

**Table 1.** Number of Different Types of De Novo Mutations.

No. De Novo Mutations	Narrow Tubes				Thick Tubes				Total
	sh01	sh02	sh03	sh04	sh01	sh02	sh03	sh04	
Total	25	31	20	55	15	27	30	97	300
Single-nucleotide variants	25	29	20	54	12	26	30	93	289
Indels	0	2	0	1	3	1	0	4	11
Categorized by fate									
Never reached frequency of 70%	8	3	0	17	3	4	4	22	61
Reached frequency of 70% but then lost	3	2	0	12	1	0	2	5	25
Fixed	14	26	20	26	11	23	24	70	214
Categorized by type									
Nonsense	0	0	0	0	0	1	1	1	3
Nonsynonymous	1	6	5	10	3	4	5	11	45
Synonymous	1	2	2	2	0	0	3	6	16
Frameshift	0	0	0	0	1	1	0	1	3
Intronic	1	2	2	3	0	0	1	5	14
Other noncoding	22	21	11	40	11	21	20	73	219

**Fig. 4.**
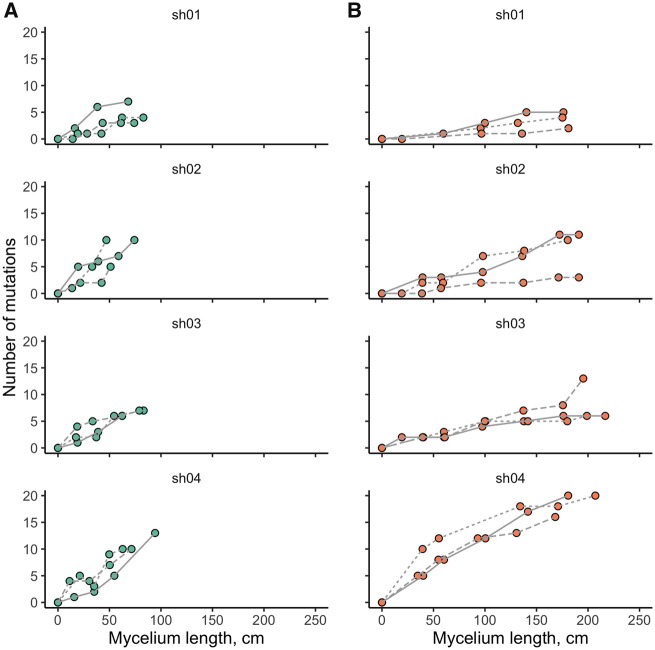
Accumulation of mutations during the growth of the mycelium. Number of mutations that have reached 70% frequency in sequenced samples are shown. Replicas are displayed with different line types. (*A*) Narrow tubes. (*B*) Thick tubes.

By the end of the experiment, between 2 and 29 mutations have reached frequencies over 70% in each line, with the mean value of nine mutations. The dynamics of this accumulation is shown in figure 4. We saw no significant change in the mutation rate over the course of mycelial growth (ANOVA test, *P* value = 0.084; supplementary fig. S2, Supplementary Material online).

**Fig. 5.**
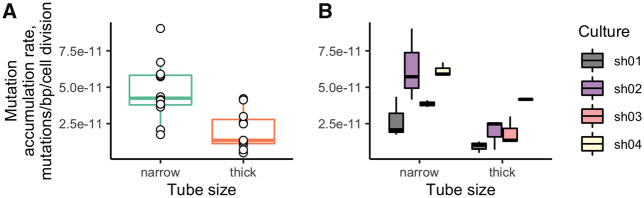
Mutation accumulation rates in narrow and thick tubes (*A*) and for individual founding cultures (*B*).

We used the mean cell length estimates of 163 µm in narrow tubes and 165 µm in thick tubes to estimate the rate at which new mutations fix in a growing mycelium per cell division of linear growth. This rate in the narrow tubes (4.99×10^−11^ substitutions/nucleotide/cell division, 95% CI: 3.62×10^−11^–6.36×10^−11^) was more than twice as high as that in the thick tubes (2.04×10^−11^, 95% CI: 1.14×10^−11^–2.93×10^−11^; ANOVA test, *P* value = 4.82×10^−5^) (fig. 5*A*). The mutation rate differed significantly between founding cultures (ANOVA test, *P* value = 0.0024) (fig. 5*B*).

## Discussion

*Schizophyllum commune* is a mycelial fungus and can grow through distances of the order of several meters, occupying whole tree trunks. One can expect *S. commune* to have some mechanism that will minimize the number of mutations accumulated during vegetative somatic growth in order to reduce the per-generation mutation rate. Both “fidelity” and “economy” mechanisms of this reduction are well-known for mammals (Kong et al. 2012; Jónsson et al. 2017) and have been recently reported for plants (Watson et al. 2016; Milholland et al. 2017) and fungi (Anderson et al. 2018, see our analysis of their data in supplementary fig. S2, Supplementary Material online, and Hiltunen et al. 2019). If *S. commune* were employing an economy mechanism similar to that found in several species with extensive vegetative growth, this would likely lead to a slower-than-linear accumulation of mutations with its growth.

In our experiment, however, mutations accumulated linearly with the number of cell divisions, so that the number of mutations was proportional to the mycelium length (fig. 2 and supplementary fig. S2, Supplementary Material online). This is what allows us to report just a single per-cell-division mutation rate for a mycelium. The mutation accumulation rate varied both between the lines and the tube sizes. Assuming that the process of mycelial growth in nature, as well as on a Petri dish, is better represented by growth in thick than in narrow tubes, we estimate the mutation accumulation rate at 2.04×10^−11^ mutations/nucleotide/cell division, or 1.24×10^−7^ mutations/nucleotide/m.

This estimate is broadly consistent with that obtained by Baranova et al. (2015). In that work, the per-generation mutation rate during growth on a Petri dish was estimated as 2×10^−8^ mutations/nucleotide/generation. Although the exact amount of mycelial growth between generations was not measured in that experiment, it was roughly ∼10 cm, giving the mutation accumulation rate of ∼2×10^−7^ mutations/nucleotide/m, which is similar to our result. It is hard to compare per-cell-division estimates of mutation rates obtained in different studies, as the number of cell divisions is usually unknown. Still, the mutation rate per unit linear growth in *S. commune* seems high. In oak, a comparison of parts of the same tree yielded the mutation rate estimate of ∼3.3×10^−10^ mutations/nucleotide/m (Schmid-Siegert et al. 2017), or ∼3.3×10^−9^ mutations/nucleotide/generation for an oak 10 m high. The per meter mutation rate in *A. gallica* is <5×10^−10^ mutations/nucleotide/m (Anderson et al. 2018). The per mitosis mutation rate in *M. oreades* fungus was found to be approximately one order of magnitude lower than that in *S. commune* (Hiltunen et al. 2019).

Although higher than in previously studied fungi and plants, the per-cell-division mutation accumulation rate in our study is lower than the somatic mutation rates in humans and mice, being closer to their germline mutation rates. In Milholland et al. (2017), the median germline mutation rates were estimated at 3.3×10^−11^ and 1.2×10^−10^ mutations per nucleotide per mitosis for humans and mice, respectively, whereas the somatic mutation rates (in fibroblasts) were estimated at 2.66×10^−9^ and 8.1×10^−9^.

Even though the per mitosis mutation rate in *S. commune* appeared to be quite moderate, the linear scaling of the number of accumulated mutations with distance may result in very large per-generation mutation rates if the mycelium growth spans large distances. If the mutations continue to accumulate linearly, a distance between fruiting bodies of ∼1 m can result in a per-generation mutation rate of the order of 10^−7^ substitutions/nucleotide, which is an order of magnitude higher than that in any species known (Lynch et al. 2016); and if this distance is larger, this rate can be even higher.

Such a high per-generation mutation rate might contribute to the extreme genetic diversity of *S. commune*. In addition, if the variability in mycelial length between fruiting bodies in *S. commune* is high, which is observed in other basidiomycetes (Anderson et al. 2018), linear accumulation of mutations may result in high variability of the per-generation mutation rate between parent–offspring pairs. Moreover, this puts previous estimation of *S. commune N*_e_ (Baranova et al. 2015) on the high end of the spectrum. If *S. commune* is, indeed, characterized by both high *N*_e_ and high per-generation mutation rates, this would imply that a high mutation rate does not need to be explained through inefficient selection in small populations (Lynch et al. 2016). Still, Xu et al. (2019) observed a low mutation rate in a duckweed *Spirodela polyrhiza* which has high *N*_e_. Thus, it is not clear if there is any causal connection between evolution of mutation rates and the strength of random drift, and this issue warrants further study.

The mutation rate differs strongly between founding cultures, and these differences are consistent between replicas (fig. 5), implying that they are at least partly determined by the genotype of the fungus. The rate in the culture from the Russian population (sh04) was larger than those in cultures collected from North American populations (sh01, sh02, sh03). This is unexpected, since the genetic diversity in the Russian population is lower than that in the North American populations (Baranova et al. 2015). The differences in diversity levels between the two populations are therefore not explainable by their different mutation rates per unit length, and may instead arise from differences in other factors such as effective population size or length of mycelia.

The rate at which mutations accumulate can be affected by selection discriminating between the growing hyphae. As selection is expected to be more efficient in larger populations (Kimura 1983), we expect its effect to be more pronounced in thick than in narrow tubes. Our data provide some evidence for such selection. First, the mutation accumulation rate is lower in thick tubes than in narrow tubes, consistent with negative selection ridding the population of some of the hyphae carrying deleterious mutations in thick tubes. Second, the mycelium growth rate decreases over the course of the experiment in the narrow tubes, consistent with accumulation of deleterious mutations in them that decrease the growth rate; but it remains constant in thick tubes, consistent with negative selection purging these mutations. Third, the d*N*/d*S* ratio among the accumulated mutations in thick tubes (but not in narrow tubes) appears to be <1, although the difference is not statistically significant. If such selection indeed operates in nature, then the actual per-generation number of mutations distinguishing the parental and offspring individuals of *S. commune* can be shaped not just by the mutation rate and the number of cell divisions, but also by the extent of competition between hyphae within a mycelium. Selection between germ line cell lineages is not unprecedented and has been observed before, for example, as competition between sperm lines in multiple species including humans, other mammals, and birds (Ramm et al. 2014) and purifying selection reducing mitochondrial heteroplasmy in mammalian female germ lines. This selection is an interesting field for further research.

## Materials and Methods

### Cultivation and Preservation

Cultures were cultivated on solid medium (beer wort Maltax10–25.6 g, water—1 l, agar—40 g) in the light at room temperature. Collected samples and founding cultures were stored at 4 and −20 °С.

### Cell Size Measurement

Founding cultures were separately cultivated in thick and narrow (2 replicates) tubes until mycelia reached length of 5 cm. Apical mycelia were sampled and longitudinal sections were prepared. Length of apical cells was measured using Altami Bio 1 microscope in transmitted light using 40×/0.65 objective, U3CMOS05100KPA camera, and ToupView 3.7.5 ToupTek Photonics software with 0.1 µm precision.

### Whole-Genome Sequencing

Before DNA extraction, samples of mycelium were first grown in liquid medium (beer wort Maltax10–8 g, water—1 l) on shaker to reach sufficient mass, and then were lyophilized. DNA was extracted using the CTAB method (Doyle and Doyle 1987). Libraries were prepared using NEBNext Ultra II DNA Library Prep Kit for Illumina with 5 PCR cycles (Accel-NGS 2S Plus DNA Library Kit with 6 PCR cycles) and sequenced on Illumina HiSeq2000 platform with 127-bp pair-end reads. Two biological replicates were sequenced independently for samples sh01–sh39.

### De Novo Genome Assembling and Annotation

Although a *S. commune* reference genome is available (Ohm et al. 2010), it is difficult to map reads from other *S. commune* individuals onto it due to extreme genetic diversity (Baranova et al. 2015). Thus, we obtained de novo genome assemblies for each founding culture. Pair-end reads were trimmed using Trimmomatic (Bolger et al. 2014) with options (ILLUMINACLIP:adapters: 2:30:10 LEADING: 3 TRAILING: 3 SLIDINGWINDOW: 4:20 MINLEN: 36). De novo genome assemblies were obtained using SPAdes (Bankevich et al. 2012) (with -only-assembler option). Assemblies were filtered of contamination using Blobology (Kumar et al. 2013). We aligned our assemblies and reference genome using Lastz (Harris 2007), removed overlapped regions using single_cov2 program from Multiz package (Blanchette et al. 2004), and used the existing annotation of the reference genome *S. commune* H4-8 v3.0 (Joint Genome Institute, http://genome.jgi.doe.gov/Schco3/Schco3.home.html, last accessed April 8, 2020) to annotate coding sequences. Assembly and annotation statistics are presented in supplementary table S3, Supplementary Material online.

### Variant Calling

Pair-end reads trimmed using Trimmomatic were mapped onto corresponding reference assemblies using bowtie2 (Langmead and Salzberg 2012). Only reads with properly mapped pair and with mapping quality 42 were kept. Duplicate reads were removed using Picard Tools (Broad Institute, available from: http://broadinstitute.github.io/picard/, last accessed April 8, 2020). De novo single-nucleotide mutations in experimental lines were called as follows. First, all positions with at least one read supporting the nonreference base were listed, and a total of 32,280 positions were obtained. At these positions, we called variants that had the following properties: 1) at least in one sample, coverage in the 10–90% range and alternative variant frequency >30%, or coverage in the 15–85% range and alternative variant frequency >20% (13,962 variants); and 2) not supported by any read in the reference sequence (289 variants). For these variants, we assessed their frequencies in all samples. For samples sh01–sh39 variant frequency was calculated as mean between two sequenced replicas. Short indels were called using samtools mpileup and freebayes software, with the same filters as described above applied. List of sequenced samples along with SRA IDs, mean coverage, and corresponding mycelium lengths are presented in supplementary table S3, Supplementary Material online. Detected de novo mutations are listed in supplementary table S2, Supplementary Material online.

### d*N*/d*S* Ratio and Expected Distributions of the Number of Nonsynonymous and Coding Mutations

d*N*/d*S* ratio was calculated using codeml program from PAML software (Yang 2007) with the following options: runmode = 0, seqtype = 1, CodonFreq = 2, clock = 0, model = 0, NSsites = 0, icode = 0, fix_kappa = 0, kappa = 2, fix_omega = 0, omega = 2, fix_alpha = 1, alpha = .0, Malpha = 0, ncatG = 4.

### ANOVA

To see how mutation rate correlates with time, we used a two-way ANOVA with genotype and tube size as a categorical fixed effects and mycelium length (which reflects time) as a continuous predictor. To see how mutation rate correlates with tube sizes and founding cultures, we used a two-way ANOVA with genotype and tube size as categorical fixed effects. To check that average sample coverage does not correlate with the inferred mutation rates, we also included mean coverage as a covariate in both ANOVA tests and saw no correlation between coverage and mutation rate.

## Supplementary Material

Supplementary data are available at *Molecular Biology and Evolution* online.

## Supplementary Material

msaa083_Supplementary_Data

## Notes

Genome sequence data have been deposited at DDBJ/ENA/GenBank under accessions WSER00000000, WSEQ00000000, WSEP00000000, and WSEO00000000, with the respective BioSample accessions SAMN13531015–SAMN13531018, under BioProject PRJNA591314. The versions described in this article are versions WSER01000000, WSEQ01000000, WSEP01000000, and WSEO01000000. Sequencing data have been deposited at the SRA with accession numbers SRR10520811–SRR10520694, under the same BioProject.
