# Supplementary material for: Rapid Accumulation of Mutations in Growing Mycelia of a Hypervariable Fungus Schizophyllum commune
Source: Mol Biol Evol. 2020 Apr 6;37(8):2279–86. doi: 10.1093/molbev/msaa083 (PMC7403608; doi:10.1093/molbev/msaa083)
Supplement: msaa083_Supplementary_Data [file msaa083_supplementary_data.zip › msaa083-suppl_data/Supplementary Figures.pdf]

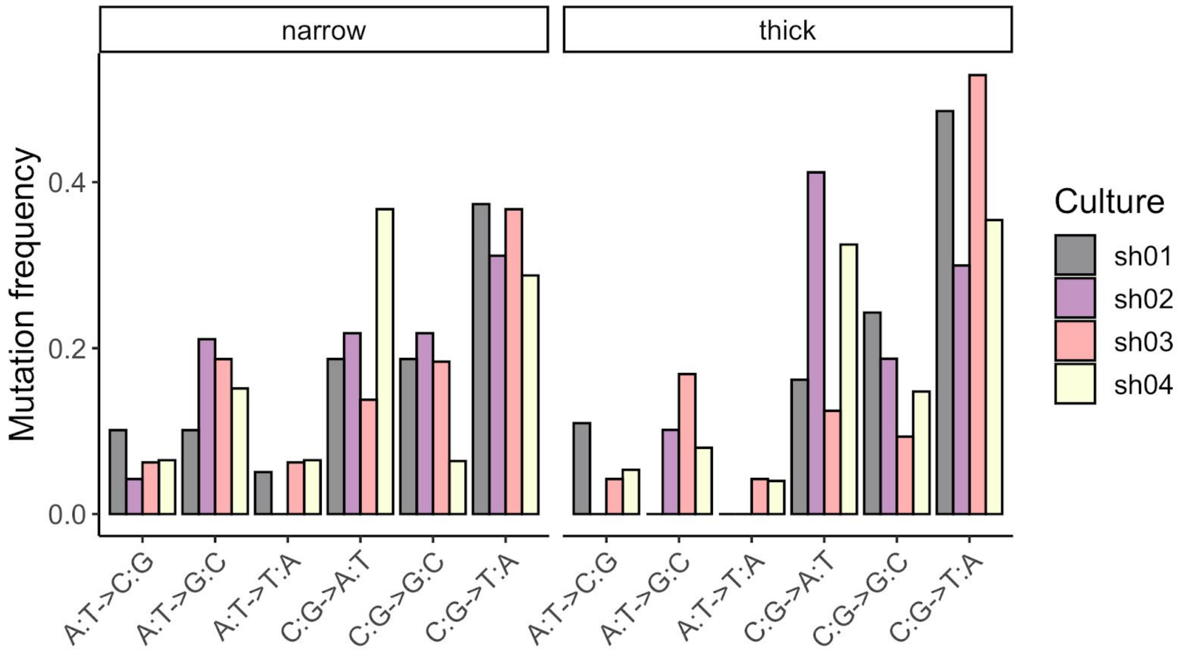

Fig. S1. Mutational spectrum for narrow and thick tubes.

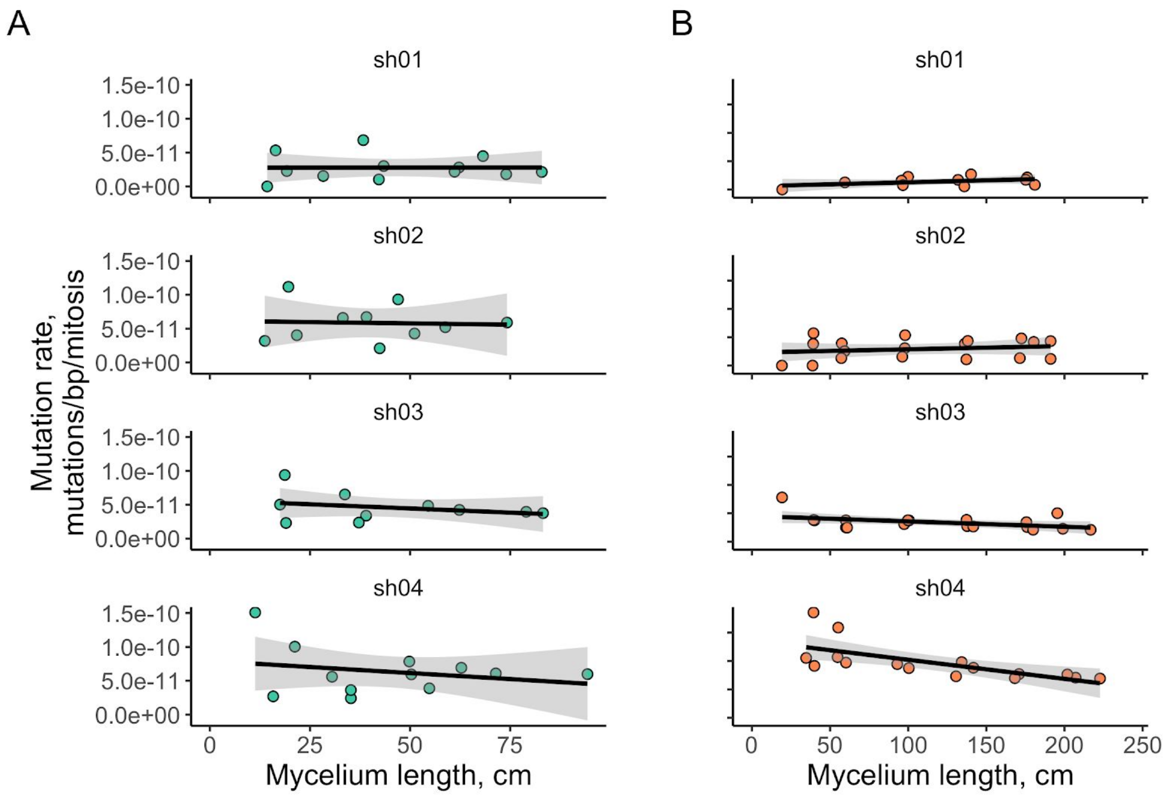

Fig. S2. Relationship between the mutation accumulation rate and mycelium length. (A) Narrow tubes. (B) Thick tubes.

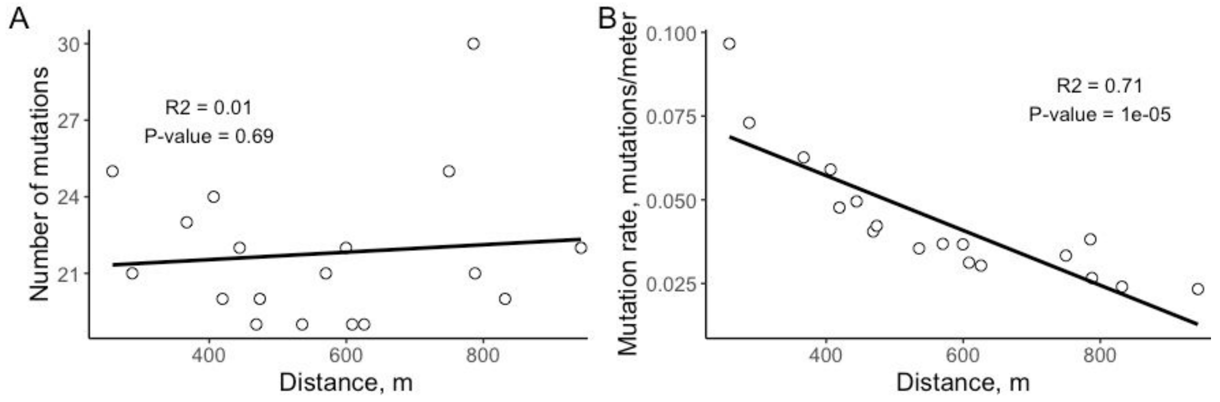

Fig. S3. Relationship between the number of mutations (A) and mutation rate, and the distance between sequenced samples in Armillaria fungus. Obtained based on data from (Anderson and Catona 2014).
